# Super-resolution imaging of T lymphocyte activation reveals chromatin decondensation and disrupted nuclear envelope

**DOI:** 10.1038/s42003-024-06393-1

**Published:** 2024-06-10

**Authors:** Jianquan Xu, Xuejiao Sun, Zhangguo Chen, Hongqiang Ma, Yang Liu

**Affiliations:** 1 Biomedical Optical Imaging Laboratory, Departments of Medicine and Bioengineering, University of Pittsburgh, Pittsburgh, PA 15213 USA; 2 UPMC Hillman Cancer Center, Division of Hematology and Oncology, Department of Medicine, University of Pittsburgh, Pittsburgh, PA 15213 USA; 3 Department of Bioengineering, Beckman Institute for Advanced Science and Technology, University of Illinois Urbana-Champaign, Urbana, IL 61801 USA; 4 Department of Bioengineering, Department of Electrical and Computer Engineering, Beckman Institute for Advanced Science and Technology, Cancer Center at Illinois, University of Illinois Urbana-Champaign, Urbana, IL 61801 USA

**Keywords:** Nanoscale biophysics, Imaging the immune system

## Abstract

T lymphocyte activation plays a pivotal role in adaptive immune response and alters the spatial organization of nuclear architecture that subsequently impacts transcription activities. Here, using stochastic optical reconstruction microscopy (STORM), we observe dramatic de-condensation of chromatin and the disruption of nuclear envelope at a nanoscale resolution upon T lymphocyte activation. Super-resolution imaging reveals that such alterations in nuclear architecture are accompanied by the release of nuclear DNA into the cytoplasm, correlating with the degree of chromatin decompaction within the nucleus. The authors show that under the influence of metabolism, T lymphocyte activation de-condenses chromatin, disrupts the nuclear envelope, and releases DNA into the cytoplasm. Taken together, this result provides a direct, molecular-scale insight into the alteration in nuclear architecture. It suggests the release of nuclear DNA into the cytoplasm as a general consequence of chromatin decompaction after lymphocyte activation.

## Abstract (web summary)

The authors show that under the influence of metabolism, T lymphocyte activation de-condenses chromatin, disrupts the nuclear envelope, and releases DNA into the cytoplasm.

## Introduction

T lymphocyte activation plays a crucial role in the adaptive immune response and the generation of cellular immunity. Prior to activation, most T cells exist in a quiescent state, becoming active only when stimulated by external pathogens. This stimulation is enabled through the engagement of T cell receptors (TCRs) with peptide major histocompatibility complex (pMHC), which forms oligomerized supramolecular activation complexes, playing a vital role in adaptive immunity. Following activation, T cells undergo extensive proliferation, differentiation and cytokine and receptor protein production, executing their function in the immune response^1,2^. This triggers a series of intracellular signaling pathways, which ultimately dictate the response of T cells.

Given the dependency of T cell signaling on the spatial organization of molecules, advanced light, and electron microscopy techniques have been extensively used to understand lymphocyte activation at nearly molecular-scale resolution^3^. These imaging techniques complement the traditional biochemical or flow cytometry assays by mapping characteristic spatial organization with molecular specificity at increasingly greater spatial and temporal resolution. For example, super-resolution imaging tools revealed molecular structure and spatial organization of various cell surface receptors on T cells that initiate the T cell signaling pathways, such as the formation of nanometer-scale protein islands formed by TCRs or signaling domains formed before and after receptor activation^4–6^.

Most studies focused on membrane ultrastructure and cell surface receptors upon lymphocyte activation. However, the nuclear architecture of lymphocytes also undergoes dramatic reorganization, accompanied by increased cell size and massive changes in epigenetic landscape and gene expression. It has been shown that lymphocyte activation induced amplification of transcriptome and histone acetylation and genome-wide chromatin decondensation^7,8^. Chromatin condensation was required for maintaining the quiescent state of T cells^9^, raising the questions regarding the impact of such dramatic changes in nuclear architecture on the structural integrity of the nuclear envelope and chromatin during antigen-specific immune cell activation.

Using single-molecule localization-based super-resolution microscopy—Stochastic Optical Reconstruction Microscopy (STORM), we extensively characterized the alterations in nuclear architecture at nanoscale resolution for T lymphocyte activation. Our super-resolution imaging and quantitative image analysis revealed that, the T cell activation resulted in significant fragmentation of genome-wide DNA folding, where more open regions of chromatin gained more accessibility to transcription factory and DNA replication machinery. Chromatin decompaction was also coupled with significant disruption of nuclear envelope, evidenced by the disrupted structure of nuclear envelope and the presence of nuclear pore complex and nuclear lamina in the cytoplasm. Remarkably, we observed the presence of double-strand (ds)DNA, forming nanoclusters in the cytoplasm, upon T cell activation. The quantity of cytosolic DNA also depended on the level of chromatin decompaction in the nucleus. Lastly, we found that disruption in nuclear architecture is metabolically dependent, with metabolic activity inhibition markedly increasing chromatin compaction and the suppression of cytosolic DNA. Together, super-resolution imaging revealed a molecular-scale disruption of nuclear architecture that facilitated the release of dsDNA into the cytoplasm in an adaptive immune response upon T lymphocyte activation.

## Results

### T lymphocyte activation results in dramatic shift in the epigenetic landscape of chromatin

**Fig. 1:**
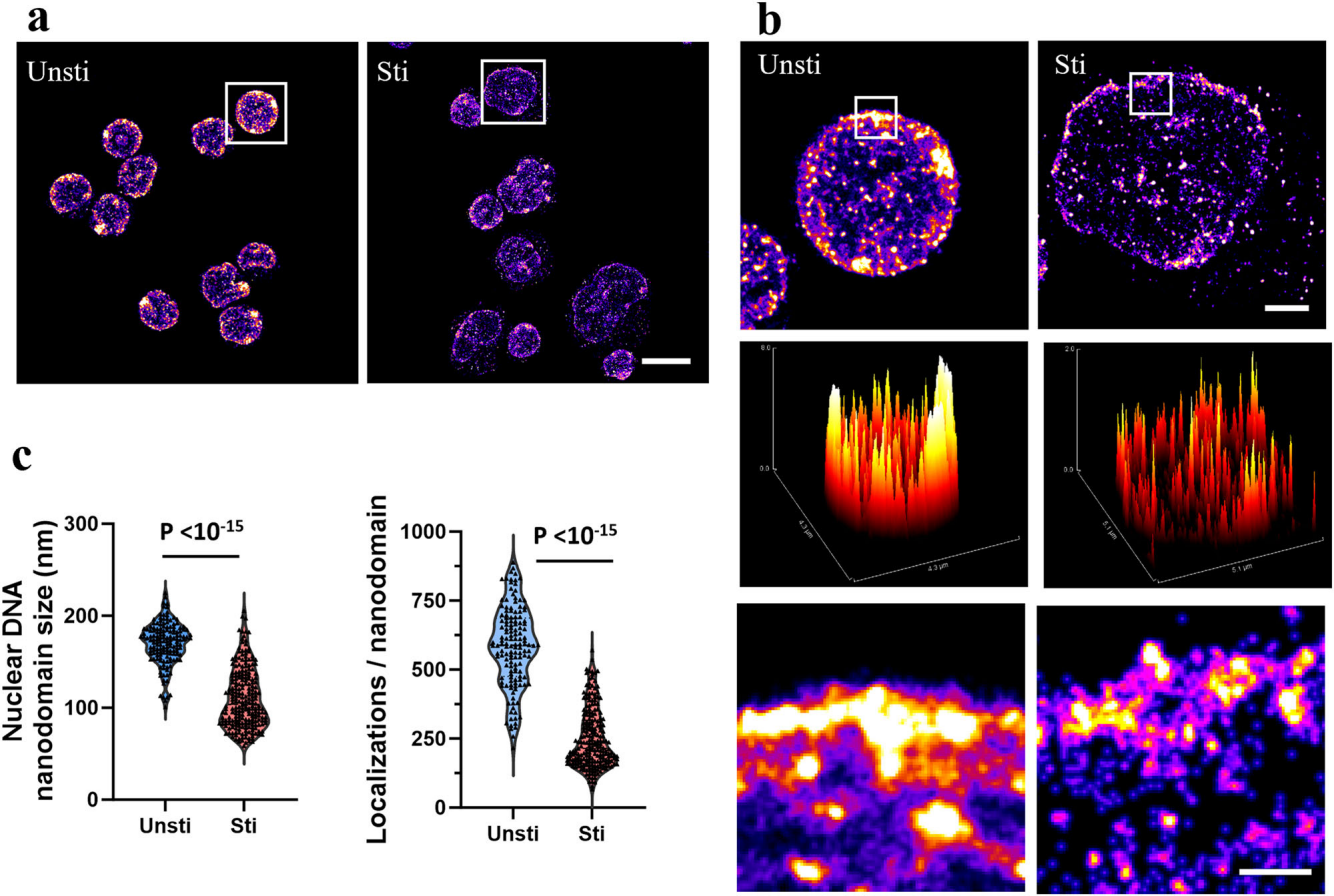
STORM imaging and quantitative image analysis of genomic DNA of T cells before and after activation. **a**, **b** Representative STORM images of genomic DNA before and after stimulation, respectively. dsDNA was labeled with small molecule Hoechst-JF646. Scale bars: 10 µm, 2 µm and 500 nm in the original and magnified images, respectively. The middle panel in **b** shows the surface plot of genomic DNA pattern of the selected cells. Photo-switching performance and resolution were shown in Supplementary Fig. S2. **c** Average nanodomain size and the number of localizations (or localized spots) of genomic DNA per cell (*n* = 232 and 151 cells, respectively).

To investigate the nanoscale structural alterations in genome-wide chromatin organization in T lymphocytes, we utilized *d*STORM imaging of dsDNA labeled with small-molecule fluorescent probes, focusing on both quiescent and activated states. Purified T cells were isolated from mouse spleen. For activation, Dynabeads™ Mouse T-Activator CD3/CD28 was employed, with the activation subsequently validated through flow cytometry (Supplementary Fig. S1). As shown in Fig. 1a, b, T cell nuclei in quiescent state are characterized by a compact and highly condensed chromatin structure, exhibiting large nanodomains of folded DNA, predominantly at the nuclear periphery, and maintaining a relatively small and round nuclear morphology. After stimulation, there was a notable enlargement of the nuclei of activated T cells^10^ (Supplementary Fig. S3a), accompanied by irregular shapes and highly decondensed chromatin structures. Chromatin organization in activated T cells exhibited fragmented compaction, forming much smaller DNA nanodomains with less accumulated DNAs within each nanodomain. These findings were supported by the significantly reduced size and the number of DNA molecules (localizations) at each DNA nanodomain in the nucleus (*p*-value < 10^−15^) in activated T cells (Fig. 1c). The nanodomain size of nuclear DNA was inversely correlated with the nuclear size (Supplementary Fig. S3b), indicating the larger nucleus has a more open chromatin structure in the activated T cells. Similar decompaction in chromatin organization was also seen in activated B cells (Supplementary Fig. S4), suggesting such chromatin decompaction is not unique to T cells but is a universal feature of lymphocyte activation.

**Fig. 2:**
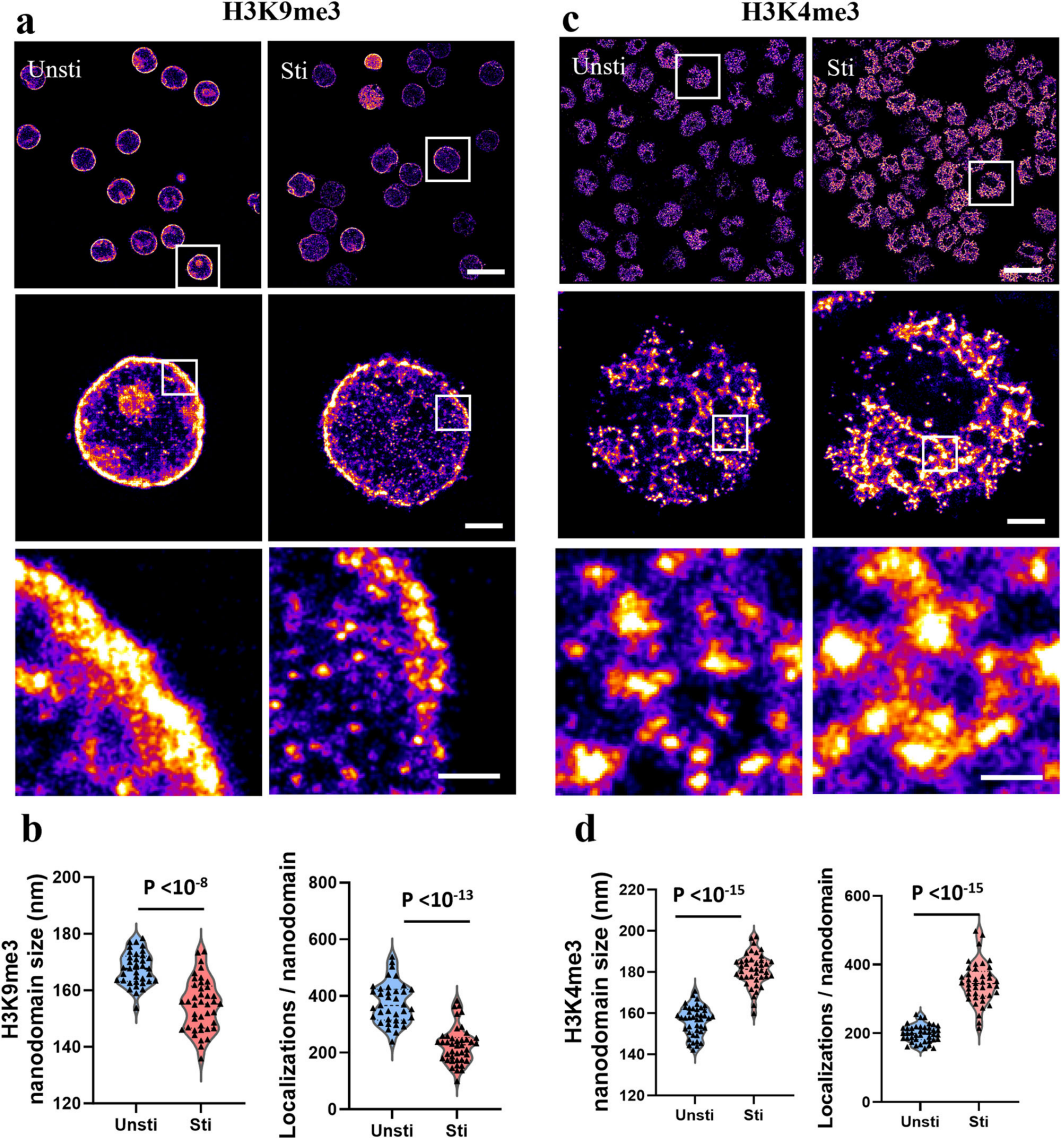
STORM imaging and quantitative image analysis of epigenetic histone modifications of T cells before and after activation. **a**, **c** Representative STORM images of genomic H3K9me3 and H3K4me3 before and after stimulation, respectively. Scale bars: 10 µm, 2 µm and 500 nm in the original and magnified images, respectively. **b**, **d** Average nanodomain size and the number of localizations (or localized spots) of H3K9me3 and for each cell H3K4me3 (*n* = 36 and 41 cells, respectively, for H3K9me3 and *n* = 42 and 41 cells, respectively for H3K4me3).

Following T cell activation, there is a dramatic shift in the epigenetic landscape of chromatin. Figure 2a–d showed significant genome-wide changes in chromatin structures and their corresponding epigenetic states following T cell activation. Specifically, the size of inactive nanodomains formed by H3K9me3 was significantly smaller with reduced accumulation of H3K9me3 marks at each nanodomain, indicating a possible decrease in the regions of inactive or repressed chromatin. Conversely, the enlargement of the active nanodomains formed by H3K4me3, accompanied by an increased presence of H3K4me3 marks in each nanodomain, suggests an expansion of the regions of active chromatin.

These data collectively support the transition of T lymphocytes to an epigenetically active state following activation. The chromatin undergoes restructuring to favor gene expression, characterized by reduced repressive marks and more prominent active epigenetic marks, and likely promotes the rapid and efficient transcription of genes vital for T cell functions in response to an activating stimulus.

### De-condensed chromatin increased DNA accessibility to transcription factory and DNA replication machinery

**Fig. 3:**
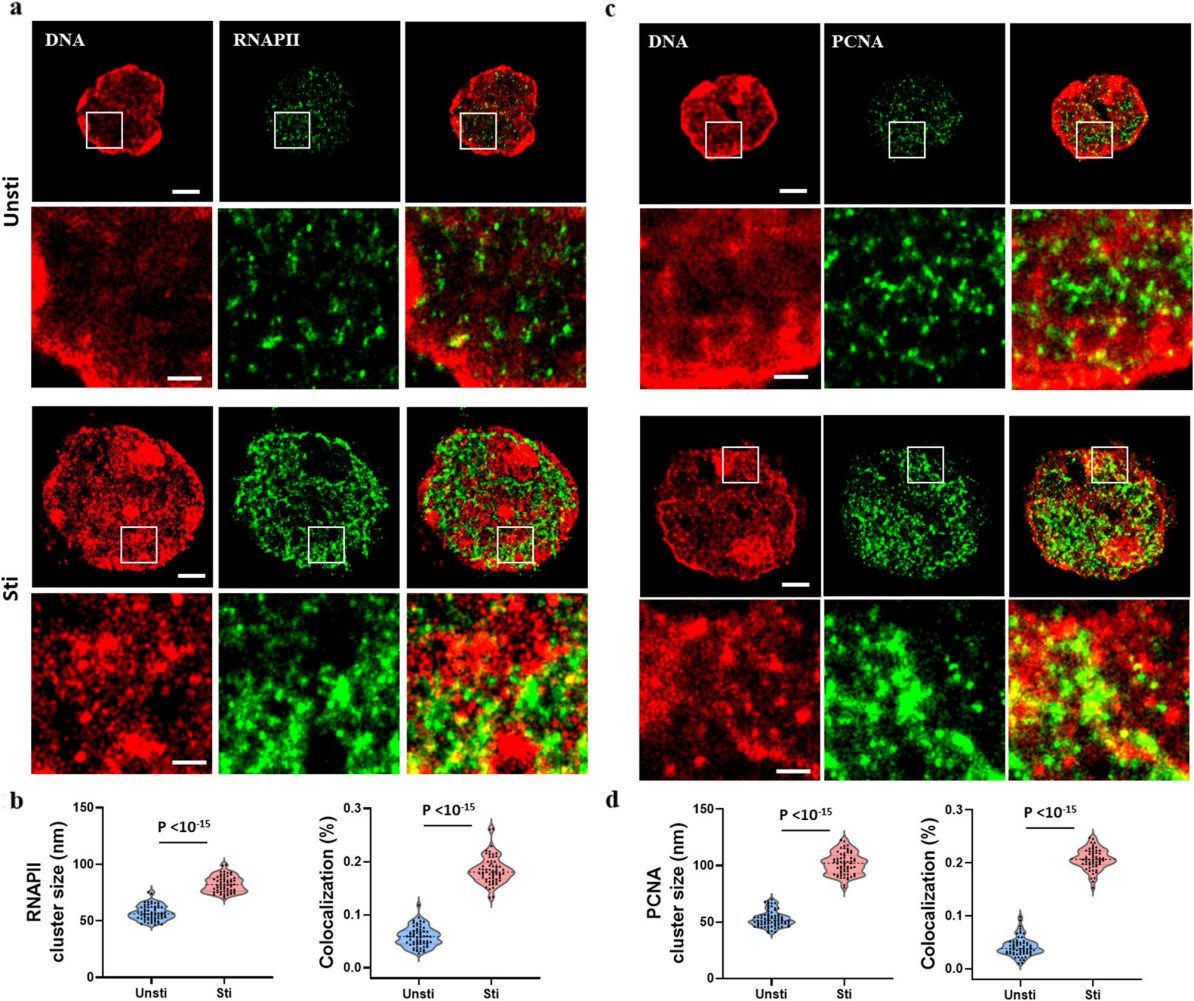
Two-color STORM images and quantitative analysis reveals spatial relationship between nuclear DNA and RNAPII or PCNA in T cells at quiescent and activated states. **a**, **c** Representative two-color STORM images of genomic DNA and RNAPII or PCNA before and after stimulation. Scale bars: 2 µm and 1 µm in the original and magnified images, respectively. **b**, **d** The average size of RNAPII or PCNA nanoclusters and degree of colocalization between nuclear DNA and RNAPII or PCNA (*n* = 63 and 58 cells, respectively for RNAPII; *n* = 74 and 60 cells, respectively, for PCNA).

T lymphocyte activation initiated by antigen-specific receptors often leads to cell proliferation and differentiation into different effector lymphocytes. It is proposed that a more open chromatin structure could increase DNA accessibility to transcription machinery, thus promoting proliferation. We next explored the spatial organization of active form of RNA polymerase II (phosphorylated RNAPII) and Proliferating Cell Nuclear Antigen (PCNA); the former being integral to active transcription factories, and the latter being crucial for DNA replication. Two-color STORM imaging between genomic DNA and RNAPII or PCNA were used to visualize the spatial relationship between chromatin organization and these two functional proteins at nanoscale resolution. As shown in Fig. 3a, b, in quiescent T cells, active RNAPII formed rather small nanoclusters located at the open regions of the chromatin, but their cluster size increased by about 44% and with 214% increase in the percentage of colocalization between RNAPII and DNA after activation. This result indicates that T cell activation markedly increased the DNA accessibility to each RNAPII transcription factory involving a significantly larger amount of phosphorylated RNAPII molecules, thus allowing for a more efficient transcription process.

Similarly, in the quiescent state, PCNA formed small nanoclusters, mainly distributed at less condensed chromatin region. After stimulation, along with the decondensed DNA folding, PCNA nanoclusters almost doubled the size relative to those in quiescent T cells, consistent with a highly boosted proliferation activity triggered by stimulation. In addition, the degree of colocalization between PCNA clusters and DNA increased by about 400% (Fig. 3c, d), suggesting its increased accessibility to DNA replication machinery after activation. The observations indicate a broader functional consequence of chromatin restructuring upon T cell activation.

Taken together, chromatin decondensation, resulting from activation, leads to an increased formation of RNAPII and PCNA clusters. This likely enhances essential cellular functions, such as transcription and replication, that are necessary for a robust immune response.

### De-compacted chromatin structure accompanied by disrupted nuclear envelope

**Fig. 4:**
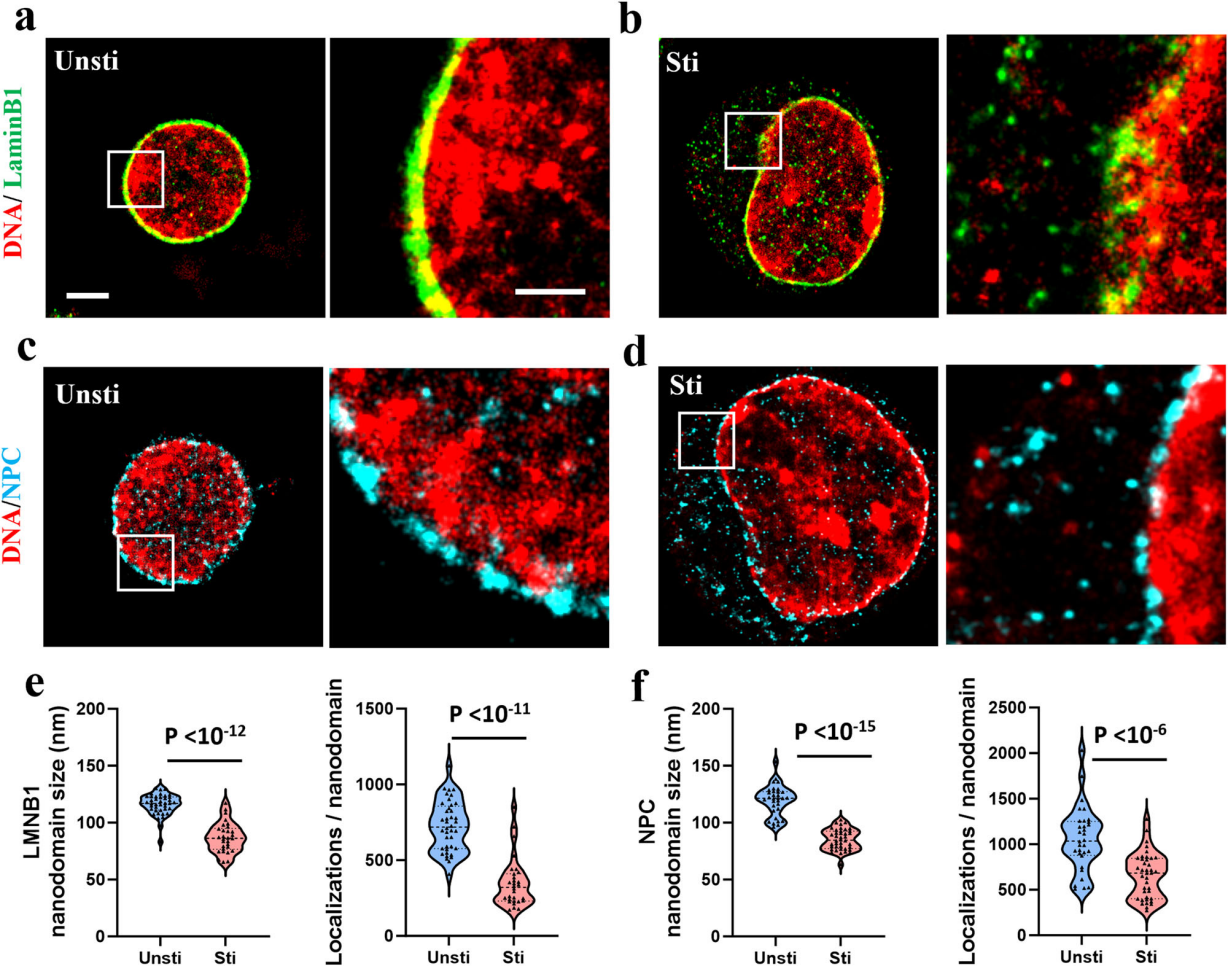
STORM imaging reveals disrupted nuclear envelope after T cell activation. **a**, **b** Two-color STORM images between genomic DNA and lamin B1 before and after activation. **c**, **d** Two-color STORM images between genomic DNA and nuclear pore complex (NPC) before and after activation. Scale bars: 2 µm and 1 µm in the original and magnified images, respectively. **e**, **f** Average nanodomain size and the number of localizations (or localized spots) of LMNB1 and NPC for each cell (*n* = 40 and 30 cells, respectively, for LMNB1; *n* = 32 and 40 cells, respectively, for NPC).

Given the established relationship between chromatin organization and the nuclear envelope^11,12^, we investigated if the observed chromatin decompaction and enlargement of the nucleus after T cell activation would affect the structural integrity of nuclear envelope. We focused our imaging on two key components of nuclear envelope—the nuclear pore complex (NPC) and the nuclear lamina protein, lamin B1. Figure 4 showed the two-color super-resolution images between DNA and lamin B1 or NPC in both quiescent and activated T cells (additional images can be found in Supplementary Fig. S5). As shown in Fig. 4a, c, quiescent T cells exhibited a well-defined and dense layer of lamin B1 and NPC closely associated with compact chromatin regions at the nuclear periphery. In contrast, the activated T cells displayed a decompacted chromatin structure, coinciding with a noticeably disrupted lamin B1 layer, with the presence of lamin B1 in the cytoplasm (Fig. 4b). The integrity of the NPC appeared even more compromised in the activated state, with a considerable portion observed in the cytoplasm (Fig. 4d). Figure 4e, f provide a quantitative analysis, showing a reduction in both size and localizations per nanodomain for lamin B1 and NPC in activated T cells, the nearest neighbor distance (nnd) of lamin B1 and NPC clusters were shown in Supplementary Fig. S6, further supporting their altered structural integrity of nuclear envelope upon activation.

These observations suggest that T cell activation not only results in chromatin decompaction but also has profound implications for the structural integrity of the nuclear envelope. The evident disruption in the key components of nuclear envelope, lamin B1 and NPC, in activated T cells implies significant structural alterations at the nuclear boundary upon T cell activation. The presence of lamin B1 and NPC in the cytoplasm further indicates a potential restructuring or reorganization of the nuclear envelope, which might facilitate specific cellular functions or responses during T cell activation. This also indicates that the nuclear pore transport may affect the T cell activation and the release of nuclear components. As the nuclear transport was blocked, the activation of T cell was nearly suspended, and we did not observe cytosolic dsDNA (Supplementary Fig. S7). This result suggests that nuclear pore transport plays an important role in the release of dsDNA in T cell activation and potentially the downstream immune signaling^1^.

### De-compacted chromatin structure and disrupted nuclear envelope linked to the release of nuclear dsDNA into the cytoplasm

**Fig. 5:**
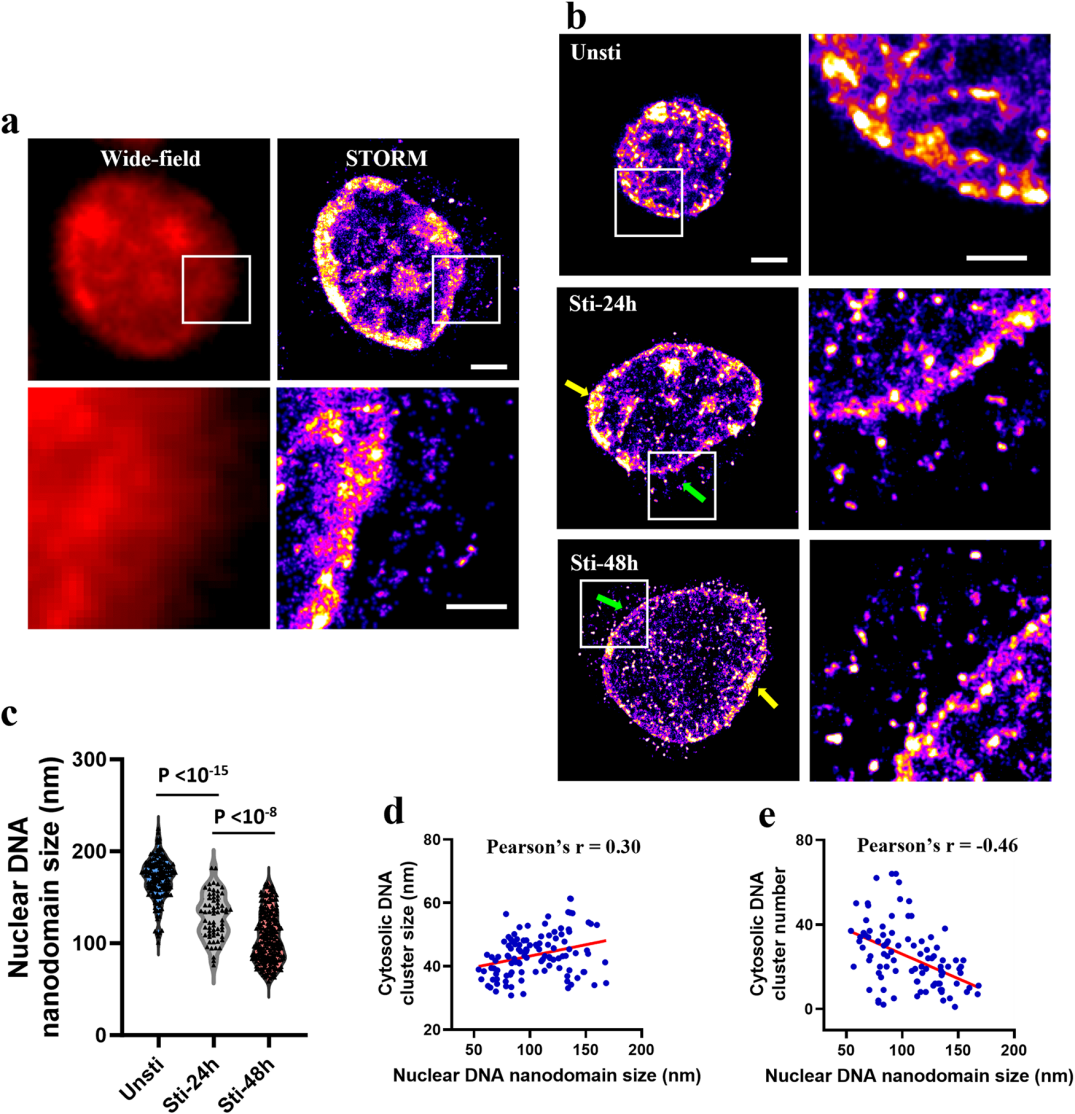
Decondensation of chromatin structure facilitates the release of nuclear dsDNA into the cytoplasm. **a** Representative conventional wide-field fluorescence image and STORM image of genomic DNA in activated T cells. **b** Representative STORM images of genomic DNA after 24 and 48 h of stimulation, respectively, as shown along the horizontal row. Green arrows indicate the regions where the cytosolic DNA is mainly located close to the disrupted chromatin region at the periphery, compared to the more compacted regions of chromatin (yellow arrows). Scale bars: 2 µm and 1 µm in the original and magnified images, respectively. **c** DNA nanodomain size in the nucleus with different stimulation time (*n* = 151, 65 and 220 cells, respectively). **d** Correlation between the size of nuclear DNA nanodomains and cytosolic DNA nanocluster size (Pearson’s *r* = 0.3). **e** Correlation between the size of nuclear DNA nanodomains and the number of cytosolic DNA nanoclusters (Pearson’s *r* = −0.46).

In addition to the marked chromatin decompaction and evident disruption of the nuclear envelope, we observed the presence of dsDNA in the cytoplasm following T cell stimulation. While the low abundance of cytosolic DNA compared to the predominant nuclear DNA rendered it nearly undetectable under a conventional microscope (Fig. 5a), the high sensitivity of STORM imaging distinctly highlighted these dsDNA nanoclusters in the cytoplasm of activated T cells. The absence of these cytosolic dsDNA nanoclusters in the quiescent T cells imaged under the identical staining and imaging conditions as those activated T cells serve as an important negative control. To further confirm that the detected cytosolic DNA is not due to non-specific binding of the small-molecule dye (Hoechst-JF646), we labeled dsDNA using immunostaining with a dsDNA antibody, a technique commonly employed for assessing cytosolic DNA^13^. As shown in Supplementary Fig. S8, the STORM images of dsDNA labeled with small-molecule dye and immunofluorescence are well merged, which further confirmed the presence of cytosolic dsDNA.

We also noticed that an increasing trend in nuclear chromatin decompaction, evident from the progressively loosened structures and diminishing average DNA nanodomain size observed at 24 and 48 h of stimulation (Fig. 5c). Interestingly, the decompaction of chromatin appeared non-uniform around the nuclear periphery. Specifically, the regions displaying greater disrupted chromatin compaction at the nuclear periphery also showed more cytosolic dsDNA nanoclusters, as illustrated by the area marked by the green arrow in Fig. 5b. In contrast, areas adjacent to more compacted chromatin, such as the region indicated by the yellow arrow, exhibited minimal cytosolic dsDNA. These data imply that the detected cytosolic dsDNA is likely released from the nucleus, from the disrupted chromatin compaction at the nuclear periphery.

We further performed quantitative analysis to evaluate the relationship between chromatin decompaction in the nucleus and the amount of dsDNA detected in the cytoplasm in the activated T cells. As shown in Fig. 5b, the cytosolic dsDNA formed heterogeneous nanoclusters, which were quantified using two parameters—the size of its nanoclusters and the number of nanoclusters detected in the cytoplasm (see Supplementary Fig. S9 for an example). As shown in Fig. 5d, e, the average size of cytosolic dsDNA nanoclusters mainly ranged from ~30 nm to 50 nm, which was positively correlated to the nanodomain size of nuclear DNA (Pearson’s *r* value = 0.30). On the other hand, the number of cytosolic DNA nanoclusters in the activated T cells was inversely correlated with the nanodomain size of nuclear DNA (Pearson’s *r* value = −0.46). These data indicate that the extent of chromatin decompaction within the nucleus has a direct impact on the release of dsDNA into the cytoplasm. Specifically, as chromatin becomes more decondensed in the nucleus, there is an associated increase in the amount of dsDNA found in the cytoplasm. Additionally, the observed correlation between the cytosolic DNA nanoclusters and nuclear DNA nanodomain size further supports this relationship. Taken together, this evidence strongly infers that the chromatin decompaction, combined with a compromised nuclear envelope, contributes to the release of dsDNA from the nucleus to the cytoplasm.

Another potential explanation for the presence of cytosolic dsDNA could be the nuclear envelope reorganization that occurs during the cell cycle. We assessed whether the detected cytosolic dsDNA was predominantly from cells transitioning into mitosis, a stage when the nuclear membrane undergoes disruption. Using Phospho-Histone H3 (Ser10) to determine the presence of activated T cells in mitosis, our data (Supplementary Fig. S10) revealed that a mere ~2% of cells were in the mitotic phase. Yet, cytosolic dsDNA was prevalent in the majority of activated cells, spanning both interphase and mitotic phase. While we recognize that some cytosolic DNA might be released during cell division, it is evident that T cell activation, leading to chromatin decondensation and nuclear envelope disruption, plays a significant role in the release of dsDNA into the cytoplasm.

### Metabolic influence on chromatin decondensation and dsDNA release after T cell activation

**Fig. 6:**
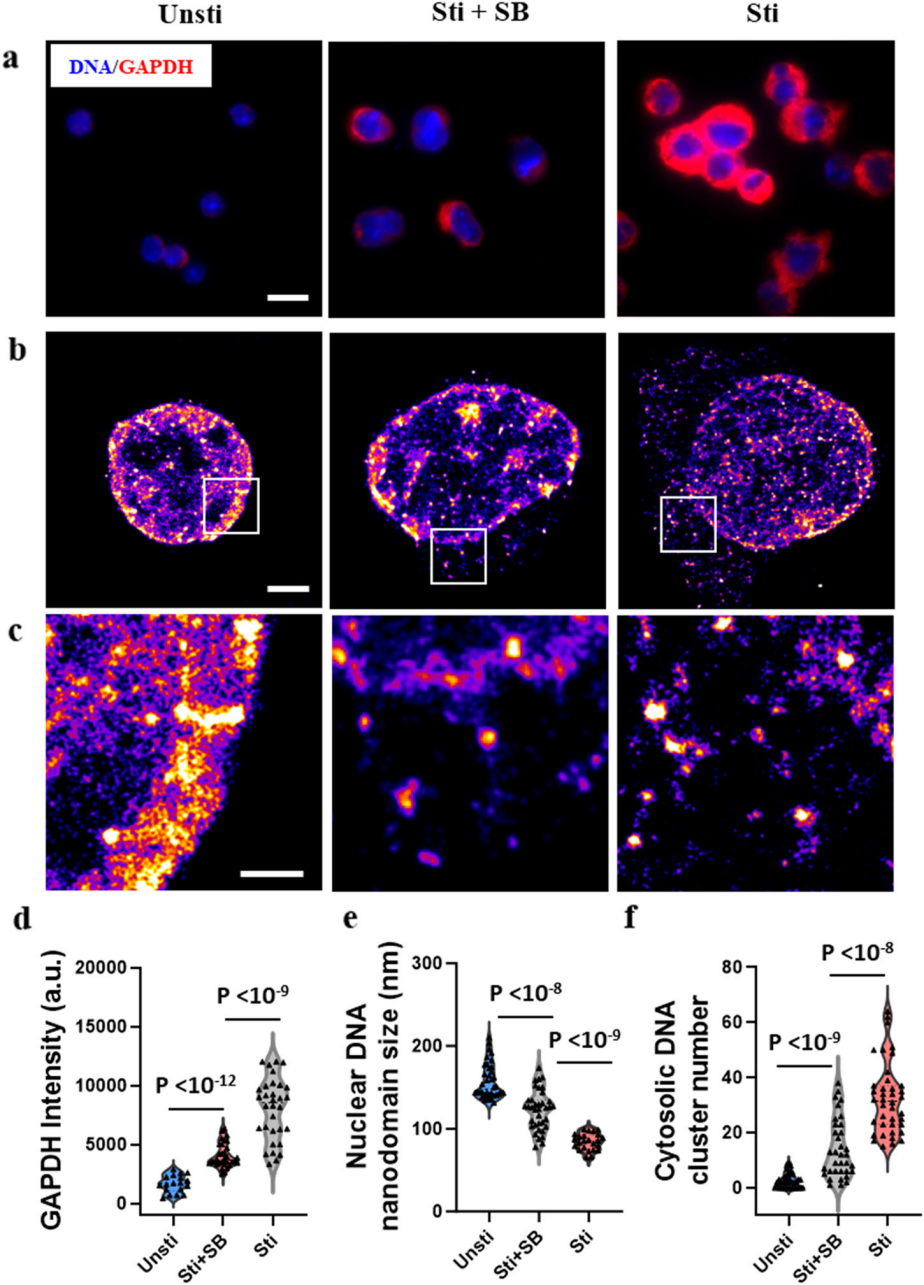
Metabolism influences chromatin decondensation and release of nuclear dsDNA. **a** Wide-field fluorescence image of GAPDH counterstained with DAPI. Scale bar: 10 µm. **b** Representative STORM images of genomic DNA of T cells without stimulation (Unsti), with SB-204990 inhibited stimulation (Sti +SB) and with stimulation (Sti). Scale bars: 2 µm. **c** Magnified images from **b**. Scale bars: 1 µm. **d**–**f** Average fluorescent intensity of GAPDH from conventional wide-field fluorescence images, average size of nuclear DNA nanodomains and number of cytosolic DNA nanoclusters from unstimulated, stimulated with metabolic inhibitor and stimulated T cells (*n* = 18, 32, and 32 cells, respectively).

During T cell activation, cells experience rapid clonal expansion, adopting an anabolic metabolism characterized by high nutrient uptake and biomass accumulation at the expense of ATP^14^. This increased metabolism supports chromatin reorganization, a process that consumes considerable energy, necessitating metabolic adjustments to meet increased energy needs^15^. We thus explored the potential influence of metabolism on chromatin decondensation and cytosolic DNA presence during T cell activation. We used SB-204990, a potent and specific inhibitor of ATP citrate lyase (ACLY) enzyme, to inhibit the metabolic activity of the T cells during stimulation. Glyceraldehyde 3-phosphate dehydrogenase (GAPDH), an enzyme important for ATP and pyruvate production, served as our metabolic marker. As shown in Fig. 6a, d, the quiescent T cells exhibited low GAPDH expression, whereas activated T cells displayed a markedly higher GAPDH levels, underscoring their highly up-regulated metabolic activities. Treatment of T cells with SB-204990 led to a notable decline in metabolic activity, supported by the reduced expression of GAPDH relative to the activated T cells.

Figure 6b, c showed the STORM images of genomic DNA in the nucleus and the cytoplasm. In cells treated with SB-204990 during stimulation, chromatin within the nucleus remained highly compact, a likely consequence of suppressed metabolic activity (Fig. 6e). Meanwhile, the inhibition also restricted the release of nuclear DNA, demonstrated by the fewer dsDNA nanoclusters observed in the cytoplasm (Fig. 6f) relative to the activated T cells. The cyclic GMP–AMP (cGAMP) synthase (cGAS) has emerged as a crucial cytosolic DNA sensor in the immune response^16^. Supplementary Fig. S11 revealed a decrease in cGAS level following SB-204990 treatment, suggesting a potential downstream effect of cGAS activation due to cytosolic DNA. Our findings underscore the interplay between T cell metabolic activity and chromatin organization. Suppressed metabolism appears to foster chromatin compaction within the nucleus, subsequently limiting nuclear dsDNA’s release to the cytoplasm during T cell activation.

## Discussion

Activation of T lymphocytes via TCR complex is an important process for T cell expansion and adaptive immune system^1^. Despite our significant knowledge on the ultrastructure of T cell receptors, the alteration of nuclear architecture in response to T cell activation is less known. Chromatin organization plays pivotal roles in immune-related functions such as transcription and DNA replication^17^. Utilizing STORM-based super-resolution imaging and quantitative image analysis, we clearly visualized the de-compaction of higher-order chromatin folding within the nucleus during T cell activation. Our findings indicate that a more accessible chromatin structure enhances interactions with transcriptional factories and replication mechanisms, thereby facilitating T cell proliferation (Fig. 3). This parallels prior observations of chromatin decompaction after B cell activation^8^, a phenomenon our super-resolution imaging of genomic DNA validates (Supplementary Fig. S4). Together, these observations support the notion that chromatin decompaction is a universal feature accompanying lymphocyte activation, preparing the cell for increased proliferation and transcription demands. Models have previously suggested the importance of physical size of the chromatin domain and transcription factor on gene activation. In line with this, smaller gene-specific transcription factors (approx. 50 kDa) have been proposed to penetrate chromatin domains, identifying target sequences, while larger transcriptional assemblies (>1 MDa) may be inhibited by condensed chromatin domain^18,19^. This aligns with our findings, suggesting that chromatin decondensation after stimulation can enhance transcription and DNA replication.

Alongside chromatin decompaction, an important and unanticipated discovery was the compromised integrity of the nuclear envelope, leading to the release of nuclear dsDNA into the cytoplasm after T cell activation. The structural organization of the nucleus plays a pivotal role in cellular functions, with the nuclear envelope acting as a crucial barrier that delineates the genomic content from the cytoplasm^20^. Our STORM imaging, as shown in Fig. 4, highlighted the presence of nuclear pores and lamin B1 in the cytoplasm after activation, indicating a pronounced alteration in the communication between the nucleus and the cytoplasm^21^. Nuclear pores are essential for nuclear-cytoplasmic transport, and the increased number of nuclear pores in the cytoplasm was previously observed alongside the reduced number of nuclear pores in the nucleus in rat cardiomyocytes^22^. This disruption of the nuclear envelope, combined with de-compacted chromatin structure, might primarily drive the release of nuclear DNA into the cytoplasm.

In mammalian cells, the cytosol is typically devoid of DNA, with genomic DNA compartmentalized within the nucleus. This compartmentalization is crucial for maintaining genome stability and DNA functionality^23^. Disruptions leading to self-DNA in the cytoplasm can initiate an innate inflammatory response^24,25^. Our super-resolution imaging revealed a correlation between nuclear dsDNA release into the cytoplasm and chromatin decompaction during T cell activation. This suggests that nuclear self-DNA release is a consequence of chromatin structural changes following lymphocyte activation. It was supported by several lines of evidence. First, as shown in Fig. 5, more fragmented chromatin structure in the nucleus is correlated with a higher number of dsDNA nanoclusters in the cytoplasm. Second, the cytosolic DNA was predominantly localized near the area of pronounced chromatin disruption. Third, disruption in nuclear envelope and fragments of nuclear envelop proteins, nuclear pore and lamin B1, were observed in the cytoplasm alongside cytosolic DNA in activated T cells. Fourth, given the 2% mitotic cells and the prevalence of the cytosolic dsDNA in the majority of activated cells, the cytosolic dsDNA is unlikely due to reorganization of nuclear membrane during mitosis (Supplementary Fig. S10). Lastly, activated B cells also exhibited nuclear DNA release into the cytoplasm, independent of specific lymphocyte activation receptors (Supplementary Fig. S4). As shown in Supplementary Fig. S12, most cytosolic DNAs fall within the size range of 30–50 nm, with some reaching the size as large as 60 nm that overlaps with nuclear DNA nanodomains. Notably, these cytosolic DNA clusters exhibit a high density of localizations, suggesting that their highly compact DNA structure is largely retained even post-release from the nucleus. This observation raises the intriguing possibility that the DNA nanodomains within the nucleus may have a robust structural integrity, enduring even after being released from the densely packed chromatin in the nucleus^19^. While cytosolic DNA frequently appears in the innate immune response, its presence due to adaptive immune response is infrequently reported. This structural disruption of chromatin compaction and nuclear envelope could serve as a cellular signal, potentially through several pathways such as innate immune signaling (cGAS/STING). Notably, a recent study aligning with our findings identified DNA threads released by activated CD4 + T lymphocytes. Although we did not explore the functional implications of cytosolic DNA besides the evidence of cGAS expression, prior research highlights its role in enhancing the inflammatory response during T cell activation^26^. Further, the results of nuclear envelope proteins seen in the cytoplasm after T cell activation is also in line with other reports that nuclear envelope disruption shares many hallmarks of cytosolic DNA and could also activate innate immune responses^27,28^.

T cell activation requires massive metabolic reprogramming to drive T cell immune response^29^. Our findings underscore the crucial role of metabolic regulation in transcription during T cell activation, highlighting its intrinsic connection to chromatin decompaction and the release of nuclear DNA into the cytoplasm. The transition from a quiescent state characterized by compact chromatin to an activated state marked by profound chromatin unfolding, reflects the metabolic shift essential for an effective immune response (Fig. 6). This metabolic-chromatin interplay directly affects the transcriptional machinery, crucial for T cell proliferation and function, by regulating access to transcriptionally active or repressive genome regions. This reorganization of chromatin is not just a consequence but a facilitator of the metabolic switch, suggesting a bidirectional relationship between metabolic processes and chromatin structure. Moreover, our data show that inhibiting metabolic activity leads to increased chromatin compaction, which in turn restricts the release of nuclear DNA into the cytoplasm (Fig. 6). This suggests that metabolic inhibitors could act as tools to modulate chromatin structure and function, offering potential therapeutic avenues for controlling T cell responses. Future studies could further elucidate the impact of metabolic state of T cells on their transcriptional landscape and how they drive the gene expression profiles necessary for their activation and function.

**Fig. 7:**
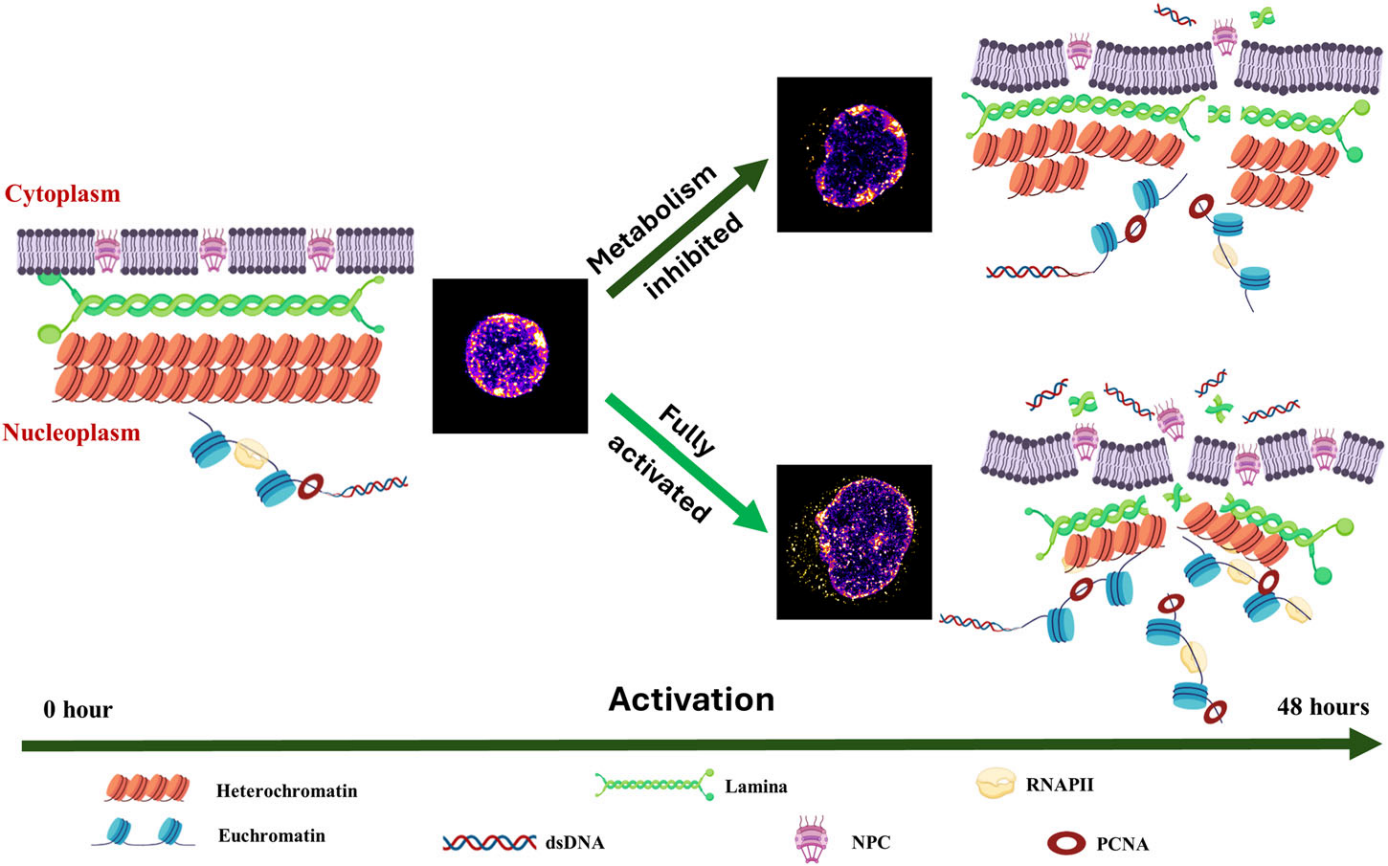
Model to depict nuclear architecture reorganization upon T lymphocyte activation. The proposed model illustrates the T cell undergoes chromatin de-condensation with increased accessibility to transcription and replication factories, nuclear envelope disruption, and the release of nuclear DNA into the cytoplasm upon T lymphocyte activation. It further illustrates the impact of metabolic activity in T cell activation, showing that metabolic inhibition leads to reduced activation and limited release of cytosolic DNA. In the visualization, nuclear DNA is represented in fire colors, while cytosolic DNA is colored in orange hot. Some elements of the figure were created with BioRender under a paid academic license.

In conclusion, our STORM-based super-resolution microscopy enabled us to observe the marked decompaction of chromatin and the disruption of the nuclear envelope during T lymphocyte activation at a nanoscale resolution. As summarized in Fig. 7, we identified the fragments of the nuclear pore complex and lamina proteins, coupled with the release of nuclear dsDNA into the cytoplasm, dependent on the extent of chromatin decompaction within the nucleus. Further, metabolism directly contributes to the extent of chromatin decompaction and subsequent nuclear envelope disruption, ultimately facilitating the release of nuclear dsDNA. Our findings offer compelling evidence that the release of nuclear dsDNA into the cytoplasm is a prevalent outcome of chromatin decompaction throughout lymphocyte activation. It also supports a synergistic relationship between metabolism, chromatin compaction, nuclear structure, and transcription in determining T cell fate.

## Materials and methods

### T/B cell purification and stimulation

Animal studies were approved by the Institutional Animal Care and Use Committee of University of Pittsburgh (Pittsburgh, PA). C57BL/6 (B6) mice were purchased from Jackson Laboratory and 8–12-week mice were used. Mice were maintained under specific pathogen-free conditions in the vivarium facility in the UPMC Hillman Cancer Center Animal Facility (Pittsburgh, PA).

### T/B cell purification

T cells (Pan T cells) were purified from mouse spleens by using EasySep mouse T cell isolation kit (19851A, Stemcell technologies) according to manufacturer’s instruction. B cells were purified from mouse spleens by using EasySep mouse B cell isolation kit (19854A, Stemcell technologies) following the manufacturer’s instructions.

### T/B cell culture and stimulation

Purified T cells at 1 × 10^6^ cells/ml were cultured with RPMI1640 (10-040-CV, Corning) media supplemented with 10% FBS and 100 µM of β-mercaptoethanol (M3148-100ML, Sigma), 1% of antibiotics (15240-062, Gibco), L-glutamine (25030-081, Gibco), MEM non-essential amino acid (25-025-CI, Coning), HEPES (25-060-CI, Corning) and sodium pyruvate (11360-070, Gibco), and stimulated by 1000 u interleukin-2 (Z00368-1, Genscript), 10 µl/ml mouse T cell activator (CD3/CD28) Dynabeads (11452D, Gibco), in 5% CO_2_ incubator for 24 and 48 h. For the metabolism inhibition, cells were incubated with 20 µM SB-204990 (SML2829, Sigma) together with the stimulation kit. For the nuclear transport inhibition, cells were incubated with 1 nM Leptomycin B (L2913, Sigma) together with the stimulation kit. B cells were cultured with the same medium and stimulated by 10 ng/ml interleukin-4 (Z02928-1, GenSript) and 10 µg/ml F(ab’)_2_ anti-IgM (115-006-075, Jackson ImmunoResearch Laboratories).

### Flow cytometry

Unstimulated or stimulated T cells were fixed by 4% paraformaldehyde. After washing with 2% FBS-PBS, cells were stained with indicated antibodies conjugated with fluorophores for 30 min, washed twice. PE-anti-CD3 (Biolegend, 100308) and Brilliant Violet 421™ anti-mouse CD86 Antibody (Biolegend, 105031) were used. Data were acquired on BD LSRFortessa X-20 cytometer and analyzed using FlowJoTM software V10 (FLOWJO).

### DNA and immunofluorescence staining on T cells and B cells

T or B Lymphocytes with or without stimulation were mounted on poly-lysine coated No. 1.5 coverslips by cytospin. The samples were then permeabilized with 0.1% Triton X-100 (Sigma-Aldrich) in PBS. To block against non-specific binding, the cell-mounted coverslips were incubated with a blocking solution containing 5% BSA (Bovine serum albumin) in PBS for 1 h at room temperature. Then the samples were incubated with Hoechst-JF646 or/and primary antibodies diluted to the optimized concentrations at 4 °C overnight: Hoechst-JF646 (1 µM, a kind gift from Lavis lab), rabbit polyclonal anti-H3K9me3 (1:600, Abcam, ab8898), rabbit polyclonal anti-H3K4me3 (1:600, Millipore, 07-473), rabbit polyclonal anti-PCNA (1:300, Cell Signaling, 13110S), mouse monoclonal anti-RNAPII (1:600, Abcam, ab5408), rabbit polyclonal anti-Lamina B1 (1:600, Abcam, ab16048), mouse monoclonal anti-dsDNA (1:300, Abcam, ab27156), rabbit monoclonal anti-Histone H3 (phospho S10) (1:300, Abcam, ab177218), mouse monoclonal (Mab414) anti-nuclear pore complex (1:300, Abcam, ab24609), rabbit monoclonal (14C10) GAPDH (1:500, Cell Signaling, 2118S), rabbit monoclonal cGAS (1:200, Cell Signaling, 31659S). Then Alexa Fluor 647 or CF568 conjugated goat-anti-rabbit/mouse secondary antibody (1:200) was applied to the samples at room temperature and incubated for 2 h in the dark. After being washed three times in PBS, the fluorescent beads (FluoSpheres carboxylate, Thermo Fisher Scientific, F8803) were deposited onto the coverslips as fiduciary markers used for drift correction. The sample was ready for STORM imaging.

### STORM imaging system

STORM images were acquired using our custom-built system on the Olympus IX71 inverted microscope frame using an oil immersion objective (UPLSAPO 100XO, NA = 1.4, Olympus) described previously^30,31^. The axial sectioning thickness in our STORM system is ~300 nm. For single-color *d*STORM imaging, the excitation laser at 642 nm (VFL-P-1000-642-OEM3; MPB Communications, Quebec, Canada) was used at a power density of ~2.5 kW·cm^−2^. The exposure time was set at 20 milliseconds and a total of 20,000 frames were used. Two-color *d*STORM imaging was conducted sequentially, where the first 20,000 frames were acquired on Hoechst-JF646 with an exposure time of 20 msec for each frame, followed by 20,000 frames of CF-568 with the same exposure time. The excitation laser of 561 nm at laser power of 0.8 kW·cm^−2^ (VFL-P-200-560-OEM1, MPB Communications, Quebec, Canada) was used for imaging CF-568-labeled targets. Drift correction was independently performed every 200 frames (or 4 s) with fluorescent nanospheres excited with 488 nm laser (DL488-150, CrystaLaser, Reno, NV) as fiduciary markers throughout each of image acquisition processes, based on our established method^30^.

### Statistics and reproducibility

The reconstruction of the super-resolution image was performed using our custom-written program written in CUDA C and described in detail in our previous publication^32^. The calculation of the size of nanodomains in the nucleus and cytoplasm was illustrated in Supplementary Fig. S9, and described in detail in our previous publication^31^, together with the calculation of degree of co-localization. Briefly, cell nuclei and cytoplasm with cytosolic DNA were first manually segmented individually to generate their binary image masks. Each nanodomain for either nucleus or cytoplasm was identified after the difference of Gaussian filtering, peak finding, followed by Gaussian mixed model (MATLAB function “fitgmdist”) to retrieve localization number and central coordinates for each nanodomain. The size of each nanodomain was calculated based on its full width at half maximum (FWHM) and the nearest neighbor distance (nnd) was calculated based on the distance between boundaries of two closest nanodomains. The statistical comparison between two groups was calculated using non-parametric Mann–Whitney U test in GraphPad Prism 9.4, and two-tailed *p*-value at 95% confidence interval was presented throughout the manuscript. and the degree of co-localization was described in our previous publication^31^.

### Reporting summary

Further information on research design is available in the Nature Portfolio Reporting Summary linked to this article.

### Supplementary information

Supplementary Information

Description of Additional Supplementary Files

Supplementary Data 1

Supplementary Data 2

Supplementary Data 3

Supplementary Data 4

Supplementary Data 5

Reporting summary

## Data Availability

Source data accomplished with the figures can be found in Supplementary Data 1–5.

## Code availability

Codes used in the manuscript are deposited in 10.5281/zenodo.11227507.
