# Supplementary material for: Super-resolution imaging of T lymphocyte activation reveals chromatin decondensation and disrupted nuclear envelope
Source: Commun Biol. 2024 Jun 10;7:717. doi: 10.1038/s42003-024-06393-1 (PMC11164909; doi:10.1038/s42003-024-06393-1)
Supplement: Supplementary file 1 — Supplementary Information [file 42003_2024_6393_MOESM1_ESM.pdf]

Supplementary Information

Super-resolution Imaging of T Lymphocyte Activation Reveals Chromatin Decondensation and Disrupted Nuclear Envelope

Jianquan Xu,[1] Xuejiao Sun,[1] Zhangguo Chen,[2] Yang Liu[1,3*]

[1]Biomedical Optical Imaging Laboratory, Departments of Medicine and Bioengineering, University of Pittsburgh, Pittsburgh, PA 15213, USA.

[2] UPMC Hillman Cancer Center, Division of Hematology and Oncology, Department of Medicine, University of Pittsburgh, Pittsburgh, PA 15213, USA.

[3] Department of Bioengineering, Department of Electrical and Computer Engineering, Beckman Institute for Advanced Science and Technology, Cancer Center at Illinois, University of Illinois Urbana-Champaign, Urbana, IL 61801, USA.

[*]Correspondence: liuy46@illinois.edu.

Supplementary Figures

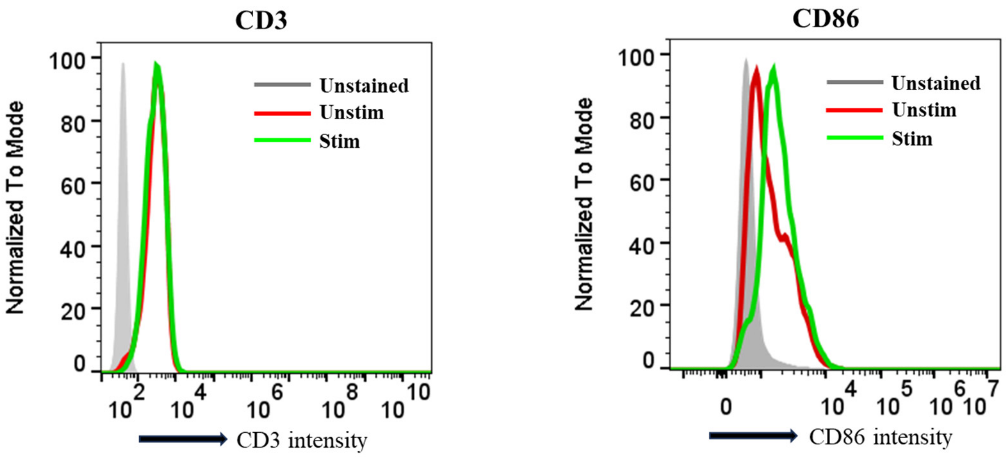

Fig. S1. CD3 and CD86 expression in T cells evaluated by flow cytometry. Results show that the stimulated (green line) and unstimulated (red line) T cells expressed the same amount CD3, while the stimulated T cells had increased expression of CD86. Gray solid areas indicate unstained T cells as negative controls. The results indicate that stimulation did not change the overall expression level of CD3, but increased CD86 expression. CD86 is activation marker for T cells, confirming that stimulated T cells were indeed activated. Debris was gated out of starting cell populations by excluding low FSC/SSC cells. Singlets were then confirmed using FSC-H/FSC-A. Cell viability was confirmed using Aqua exclusion. Cells were then gated by marker expression as described in Methods and Supplementary information. All gates were determined by fluorescence-minus-one controls, set at<0.1% positive cells for each control.

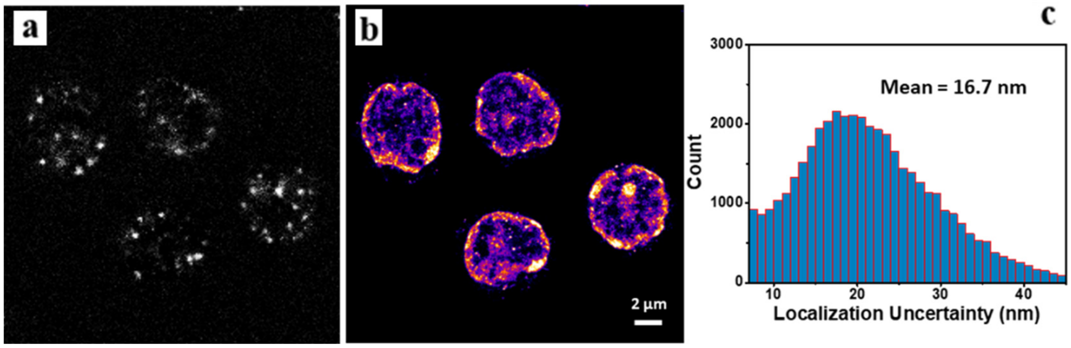

Fig. S2. Switching performance of Hoechst-JF646 for STORM imaging. (a) A representative single-frame raw image of genomic DNA (labeled with Hoechst-JF646) of lymphocyte T cells, live blinking video was shown in Supplementary Movie 1. (b) The corresponding reconstructed STORM image of DNA of T cells. (c) Histogram of localization precision (mean value = 16.7 nm) corresponding to an image resolution of ~39 nm.

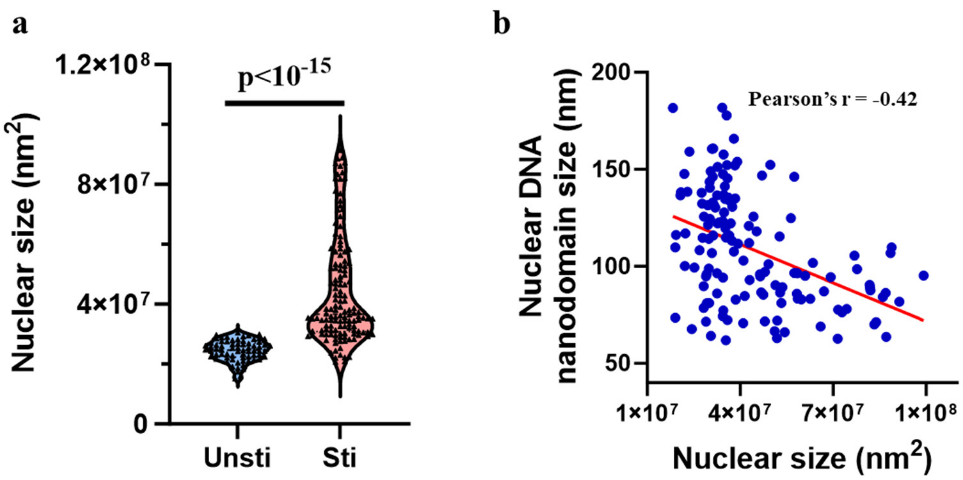

Fig. S3. (a) Nuclear area of T cells before and after stimulation. **(b)** The DNA nanodomain size in the nuclei is inversely correlated with the nuclear area in the stimulated T cells (Pearson's r = -0.42).

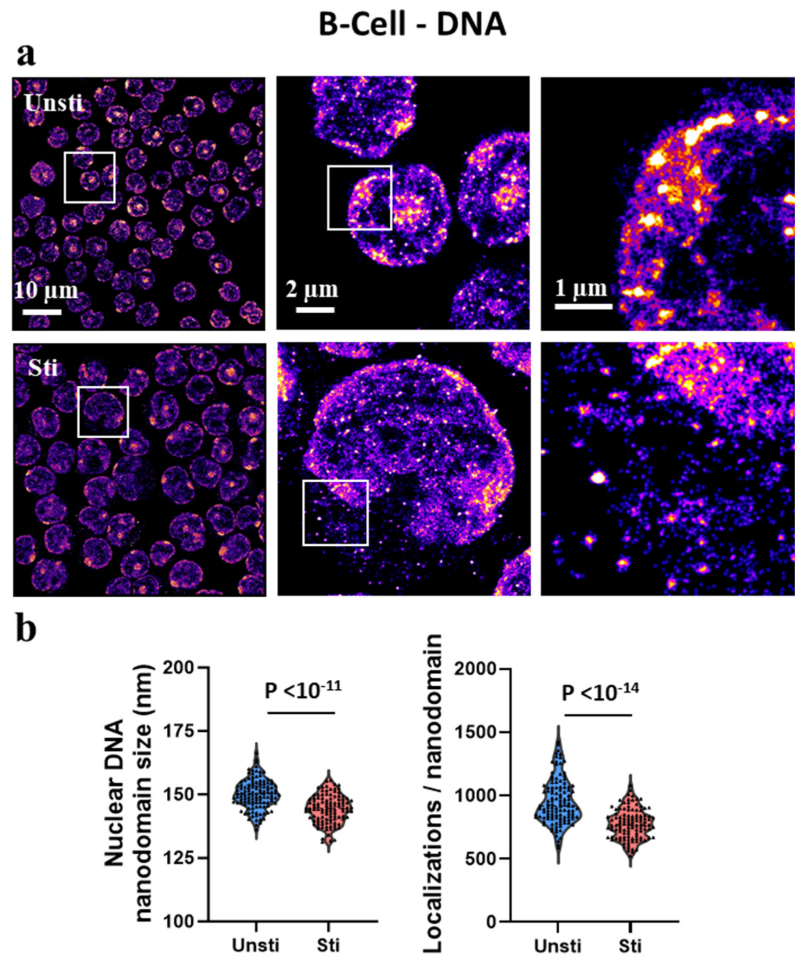

Fig. S4. Super-resolution images and quantitative analysis of chromatin structure in B cells.

(a) Representative STORM images of genomic DNA of B cells before and after stimulation. Each spot represents the average value for each cell. **(b)** Statistical analysis of the DNA nanodomain size and number of localized fluorescent emitters per cell between unstimulated (quiescent) and stimulated B cells. Approximately 100 B cells were imaged and quantified here.

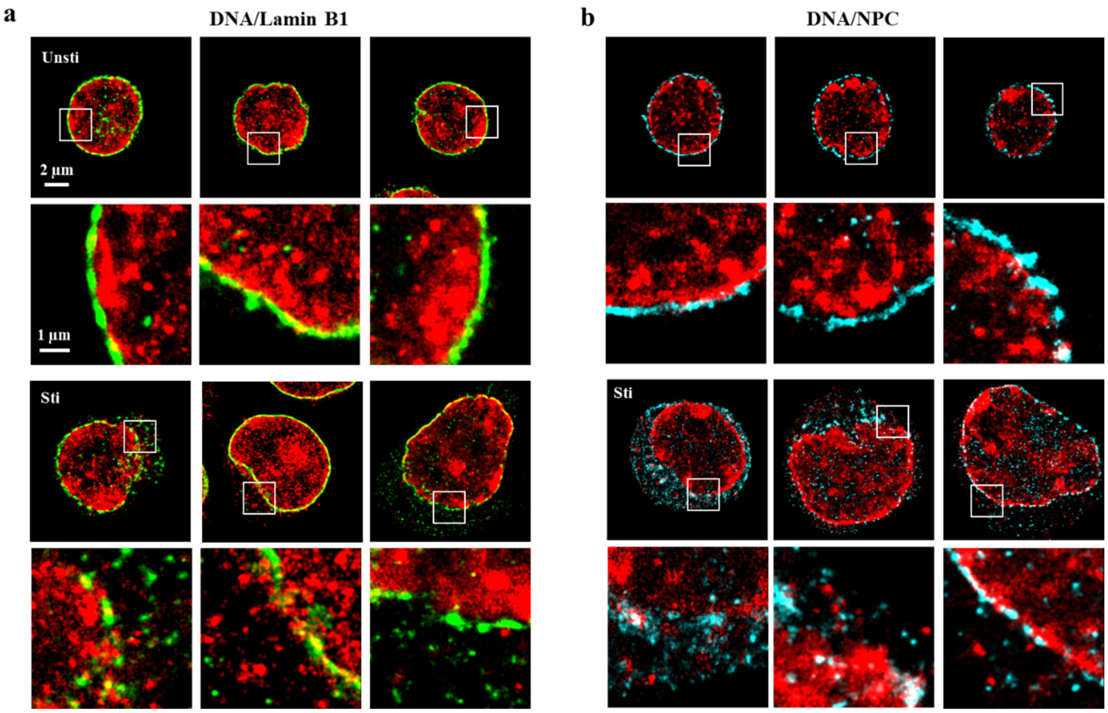

Fig. S5. Two-color STORM images between DNA and lamin B1 or nuclear pore complex (NPC).

(a) Two-color STORM images between genomic DNA and lamin B1 before and after activation. **(b)** Two-color STORM images between genomic DNA and nuclear pore complex before and after activation. These are additional images besides those shown in Figure 4 in the main manuscript.

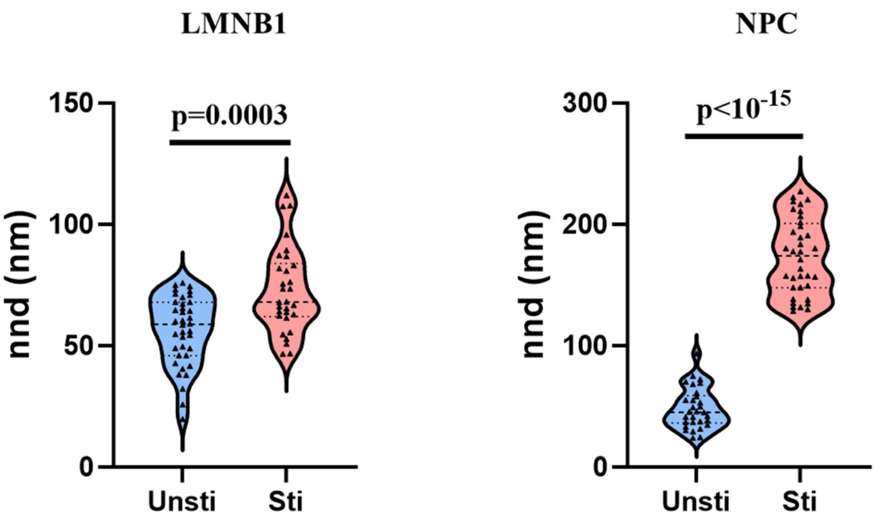

Fig. S6. The nearest neighbor distance (nnd) of nanodomains formed by lamin B1 (LMNB1, left panel) and nuclear pore complex (NPC, right panel), respectively.

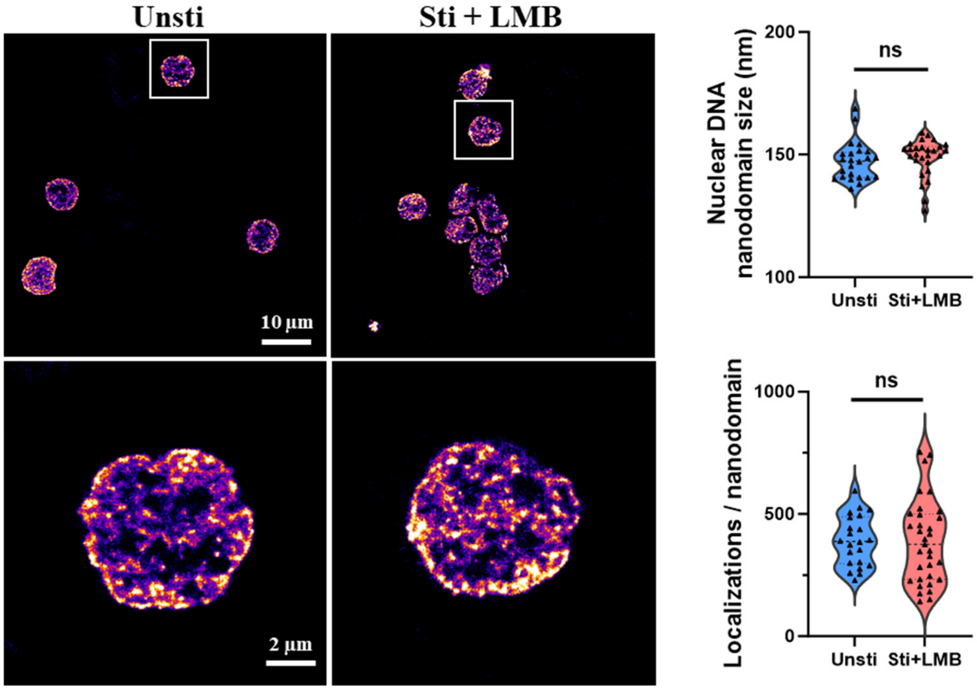

Fig. S7. Representative STORM images and quantitative image analysis on genomic DNA in the unstimulated and stimulated T cells in the presence of Leptomycin B (LMB). Cytosolic dsDNA is barely detectable from the STORM images in stimulated T cells in the presence of LMB. Chromatin structure in the nucleus also showed no significant difference between unstimulated and stimulated T cells in the presence of LMB.

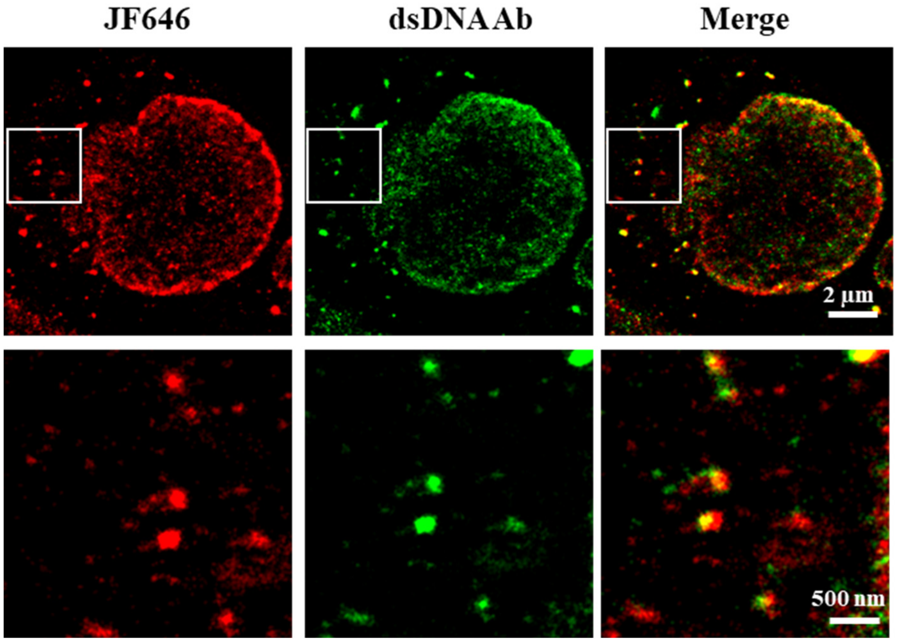

Fig. S8. Validation of dsDNA labeling with two-color STORM imaging. Two-color STORM images were obtained from dsDNA labelled with two different methods – small-molecule dye of Hoechst-JF646 and immunofluorescence against CF568-conjugated anti-dsDNA antibody.

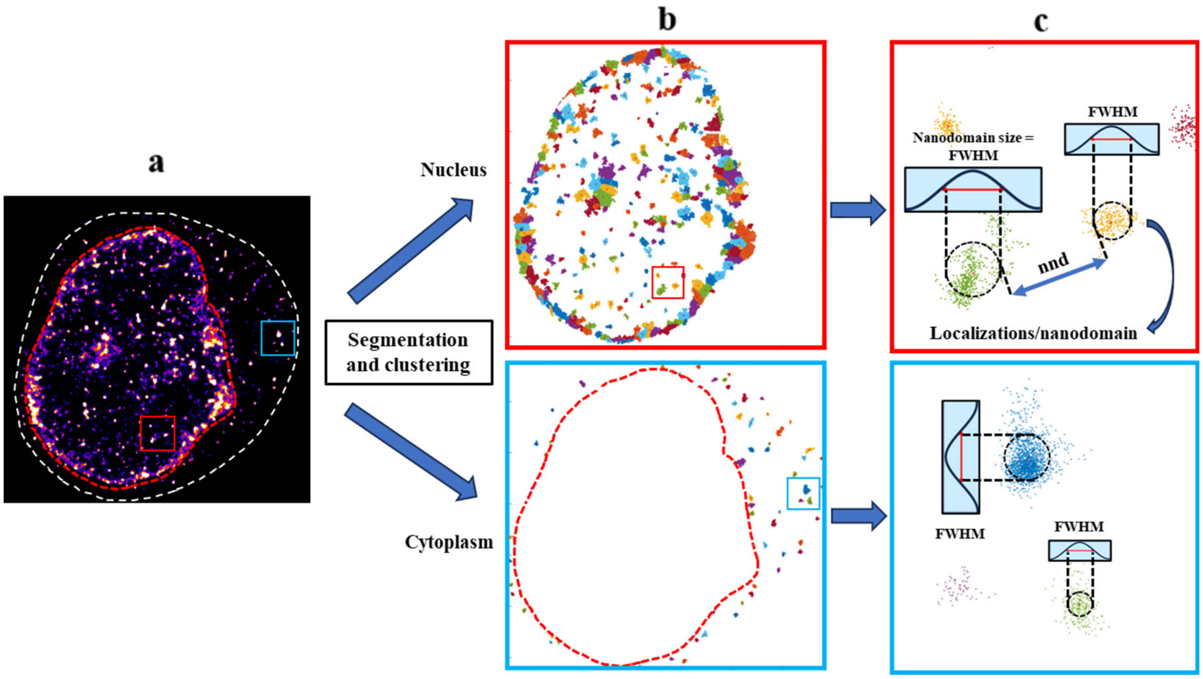

Fig. S9. Image processing pipeline to identify dsDNA nanodomains in the nucleus and cytoplasm.

(a) A representative STORM image of dsDNA in the nucleus and cytoplasm of an activated T cell. **(b)** Segmented nanodomains are marked by different colors. **(c)** The nanodomain size is defined by the full width at half maximum (FWHM) of each cluster from the regions within the boxes in (B). The nearest neighbor distance (nnd) is defined as the distance between the boundaries of two closest nanodomains.

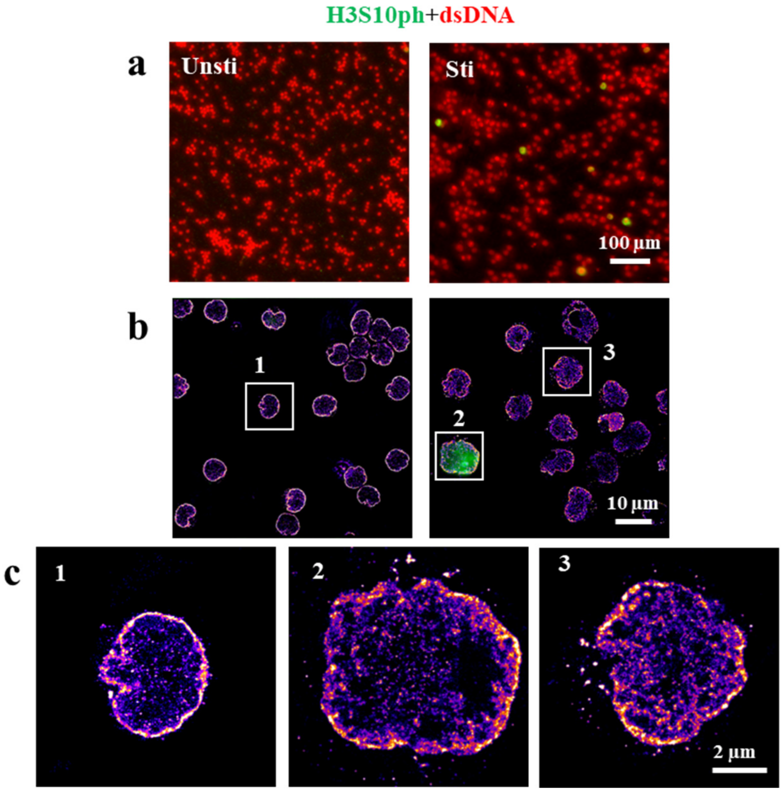

Fig. S10 (a) Wide-field fluorescence images of unstimulated and stimulated T cells stained with Phospho-Histone H3 (Ser10) and dsDNA. **(b)** STORM images of dsDNA in unstimulated and stimulated T cells co-stained with Phospho-Histone H3 (Ser10). **(c)** Zoomed regions from (b), (1) unstimulated cell, (2) stimulated cell in mitotic phase, (3) stimulated cell in interphase. Approximately 2% of activated T cells were in the mitotic phase.

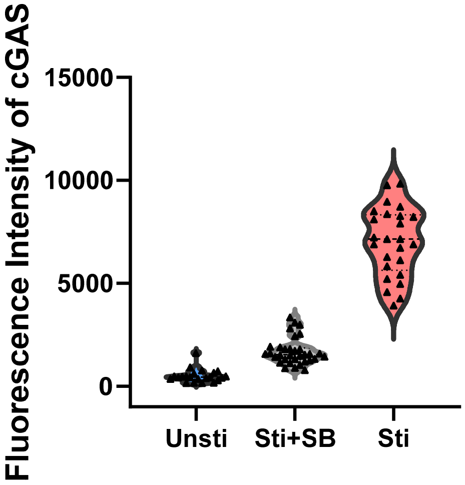

Fig. S11. Fluorescent intensity of cGAS in quiescent T cells without stimulation (Unsti), with stimulation in the presence of a metabolic inhibitor SB-204990 (Sti + SB) and with stimulation (Sti). Each point represents the average fluorescence intensity value of cGAS per cell. Over 20 cells were analyzed in each group.

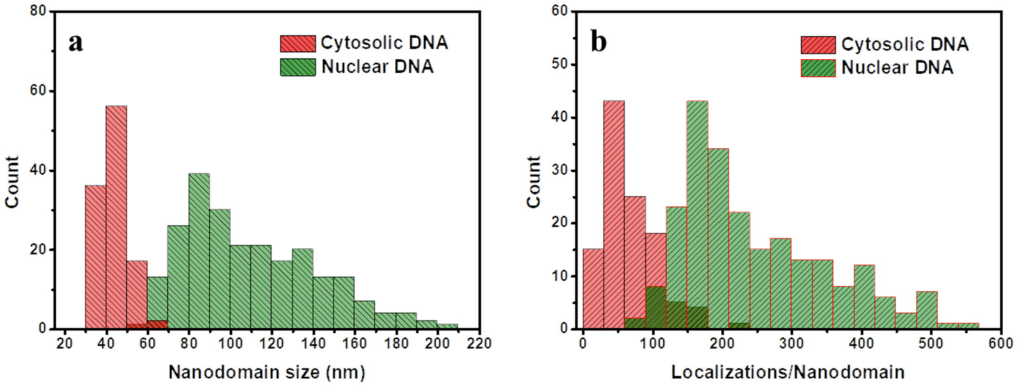

Fig. S12. Distribution of nanodomain size **(a)** and number of localizations per DNA nanodomain **(b) of** cytosolic dsDNA and nuclear DNA nanodomains.
