# Supplementary material for: Super-resolution imaging of T lymphocyte activation reveals chromatin decondensation and disrupted nuclear envelope
Source: Commun Biol. 2024 Jun 10;7:717. doi: 10.1038/s42003-024-06393-1 (PMC11164909; doi:10.1038/s42003-024-06393-1)
Supplement: Supplementary file 2 — Description of Additional Supplementary Files [file 42003_2024_6393_MOESM2_ESM.pdf]

Description of Additional Supplementary Files

File name: Supplementary Data 1

Description: The source data behind Figure 1 and 2 in the paper

File name: Supplementary Data 2

Description: The source data behind Figure 3 in the paper

File name: Supplementary Data 3

Description: The source data behind Figure 4 in the paper

File name: Supplementary Data 4

Description: The source data behind Figure 5 in the paper

File name: Supplementary Data 5

Description: The source data behind Figure 6 in the paper
